# Fear of Missing Out and Smartphone Addiction Mediates the Relationship Between Positive and Negative Affect and Sleep Quality Among Chinese University Students

**DOI:** 10.3389/fpsyt.2020.00877

**Published:** 2020-08-27

**Authors:** Li Li, Mark D. Griffiths, Songli Mei, Zhimin Niu

**Affiliations:** ^1^School of Humanities and Social Sciences, Gannan Medical University, Ganzhou, China; ^2^International Gaming Research Unit, Nottingham Trent University, Nottingham, United Kingdom; ^3^School of Public Health, Jilin University, Changchun, China

**Keywords:** trait-fear of missing out, state-fear of missing out, smartphone addiction, sleep quality, positive affect, negative affect

## Abstract

**Background and Aims:**

The widespread use of social media on smartphones has lead to the fear of missing out (FoMO) and smartphone addiction among a minority of adolescents and adults. However, few studies have investigated the impact of trait affect on sleep quality *via* FoMO and smartphone addiction. The present study examined whether FoMO (trait-FoMO and state-FoMO) and smartphone addiction mediated the relationship between positive affect (PA)/negative affect (NA) and sleep quality, and the prevalence of sleep disturbance among Chinese university students.

**Methods:**

The sample comprised 1,164 university students and they completed a survey which included the Chinese Trait-State Fear of Missing Out Scale (T-SFoMOS-C), Mobile Phone Addiction Index (MPAI), International Positive and Negative Affect Scale Short-Form (I-PANAS-SF), and the Pittsburgh Sleep Quality Index (PSQI).

**Results:**

The prevalence of sleep disturbance was found to be 15.98% among Chinese university students. The serial multiple mediation effects indicated that PA directly impacted on sleep quality, but the mediation effects of trait-FoMO and state-FoMO were not found. NA impacted on sleep quality *via* the mediation effects of trait-FoMO/state-FoMO and smartphone addiction.

**Conclusion:**

Negative affect was positively associated with poor sleep quality, which was partially mediated by FoMO and smartphone addiction among Chinese university students. Individuals with high negative affect were more likely to have high levels of FoMO and were more prone to smartphone addiction as well as experiencing poor sleep quality. These findings provide an evidence base for emotion management, prevention of smartphone addiction, and sleep improvement.

## Introduction

The phenomenon of ‘fear of missing out’ (FoMO) has become more prevalent due to social media use over past few years (1, 2). A survey from *Xinli001* (i.e., a professional psychological information and service platform in China) reported that 15.2% of respondents experienced severe FoMO (3). As “a pervasive apprehension that others might be having rewarding experiences from which one is absent” (1), FoMO is also considered as a two-dimensional construct including trait-FoMO (i.e., “a relatively stable individual characteristic”) and state-FoMO (i.e., “fear of missing out online content and interaction with others using social media”) (4).

Recent studies have identified that social media use (5–9), psychological need satisfaction (1, 10–12), and personality traits (13–17) may be considered as risk factors of FoMO. Wolniewicz et al. also reported that FoMO was most strongly associated with both problematic smartphone use and normal smartphone use (e.g., video and voice calls, text/instant messaging, email, social networking sites) relative to negative affect and fears of negative and positive evaluation (18), similar to the relationship between FoMO and other types of internet addiction (e.g., Facebook addiction, social networking site [SNS] addiction) (19–23). State-FoMO has been found to directly and indirectly impact phubbing *via* problematic Instagram use, whereas trait-FoMO has been indirectly associated with phubbing *via* state-FoMO and problematic Instagram use (24). Additionally, some studies have shown the associations between high levels of FoMO and negative outcomes, such as bad school performance, fatigue, and decreased sleep (25–28).

In March 2020, the China Internet Network Information Centre reported that the total number of Chinese internet users was 904 million, with 897 million accessing the internet *via* smartphones (99.3%) (29). Smartphone addiction has been found to be prevalent among adolescents and emerging adults in China and elsewhere in the world (30–32). The prevalence of problematic smartphone use/smartphone addiction was estimated in one study to be 21.3% among Chinese undergraduates (33).

As a form of technological addiction or one of generalized internet addictions (34–37), smartphone addiction is also described as “an inability by individuals to regulate their use of smartphones and which eventually leads to negative consequences and clinical impairment in daily life” (38). Some risk factors of smartphone addiction have been examined such as negative affect (e.g., depression, anxiety and loneliness), low Internet self-efficacy, high impulsivity, as well as narcissism (39–45). In studies of smartphone addiction, Additionally, smartphone addiction has shown demonstrable association with alcohol use disorder symptoms, specific mental health diagnoses (i.e., ADHD, anxiety, depression, and PTSD), poor scholastic performance (46), vision problems (47), driving risk (48), and musculoskeletal pain (49–52). Smartphone addiction is also associated with poor sleep quality (32, 53, 54). Most studies have also shown that smartphone addiction is often associated with social media addiction because social media use is primarily engaged in *via* smartphones (55, 56). Negative urgency has been shown to mediate the relationship between negative emotion and smartphone addiction (57). Moreover, smartphone addiction may serve as a mediator between loneliness and sleep quality (30).

Trait affect as a key personality construct has been an important concept in applied psychology (58). The positive and negative dimensions of trait affect have been defined by several scholars (59, 60). Positive affect (PA) reflects “the extent to which a person feels enthusiastic, active, and alert”, whereas negative affect (NA) is “a general dimension of subjective distress and unpleasurable engagement that subsumes a variety of aversive mood states, including anger, contempt, disgust, guilt, fear, and nervousness” (61).

Positive affect and negative affect as ambivalent emotions have been found to be associated with high levels of FoMO when using Facebook (1). Przybylski et al. developed the Fear of Missing Out Scale (FoMOS), which is a unidimensional scale for assessing trait FoMO. Individuals experiencing lower levels of general mood reported higher levels of FoMO (1). Moreover, FoMO has been found to mediate the relationship between general mood and social media engagement, as well as basic need satisfaction/life satisfaction and social media engagement (1). Riordan et al. reported that negative affect has been associated with FoMO when using *Facebook* (62). Shin et al. also reported the mediating effects of affect on the associations between personality factors and internet gaming disorder (63). In addition, envy as a specific negative affect has been shown to impact on problematic smartphone use and SNS addiction *via* FoMO (64, 65). In the interaction of Person-Affect-Cognition-Execution (I-PACE) model, Brand et al. proposed that internet-related disorders (e.g., smartphone addiction, social media addiction) were the consequence of interactions between predisposing variables (e.g., neurobiological and psychological components), moderators (e.g., coping style and internet-related cognitive biases), and mediators (e.g., affective and cognitive responses to specific stimuli in combination with reduced executive functioning) (66, 67). In the early stages of behavioral addiction, negative or positive moods as internal triggers may be perceived, which result in affective (e.g., FoMO) and cognitive responses and increase attention to stimuli and urges to behave in specific ways (e.g., urge to use smartphone, play online gaming) (67, 68).

Some studies have reported that sleep disturbances are common among Chinese university students (69, 70). A recent meta-analysis examining sleep problems and internet addiction reported a significant odds ratio (OR) for sleep problems and a significant reduction in sleep duration among individuals addicted to the internet (71). Some studies have reported that sleep quality is positively associated with NA and negatively associated with PA (72, 73). Although, some scholars argue that the relationship between sleep quality and affect may be bidirectional (74–76), previous research has also shown that both positive and negative affect can mediate the impact of expressive suppression on sleep quality (73, 77). Another recent study showed that insomnia partially mediated a significant association of interpersonal stress and FoMO with mental health (78). Moreover, the relationship between negative affect and sleep quality may also be mediated by smartphone addiction (79).

**Figure 1:**
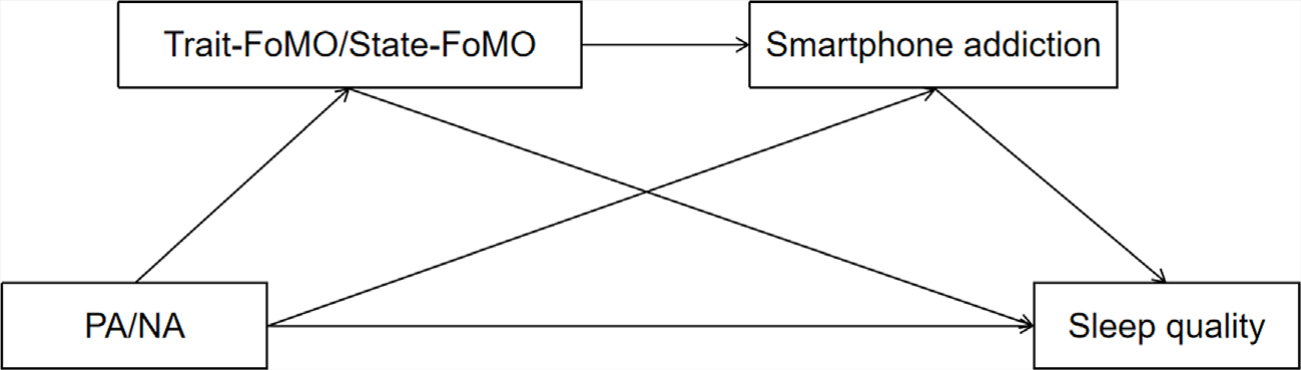
The proposed series multiple mediation model.

Based on the I-PACE theory and previous studies, the aim of the present study was to carry out a multiple mediation analysis examining PA/NA to sleep quality, in which trait-FoMO/state-FoMO and smartphone addiction were the mediator variables. It was hypothesized that a) PA/NA, trait-FoMO/state-FoMO, and smartphone addiction would be associated with sleep quality; b) trait-FoMO and smartphone addiction would mediate the association between PA and sleep quality; c) state-FoMO and Smartphone addiction would mediate the association between PA and sleep quality; d) trait-FoMO and smartphone addiction would mediate the association between NA and sleep quality; and e) state-FoMO and smartphone addiction would mediate the association between NA and sleep quality (**Figure 1**).

## Methods

### Participants

A cross-sectional study was carried out from October 2019 to November 2019. The survey was completed on the Wenjuanxing platform (www.wjx.cn). The participants comprised 1,258 students (from 17 to 25 years; mean age = 20.1 years; SD ± 1.6) by cluster convenience sampling from three universities in Jiangxi province and Liaoning province of China. However, 94 participants were excluded from the dataset owing to incomplete information. The final sample comprised 1,164 participants (656 females, 508 males) with a 92.5% response rate.

### Measures

#### Pittsburgh Sleep Quality Index (PSQI)

Sleep quality was assessed using the Pittsburgh Sleep Quality Index (PSQI) (80), which assessed sleep disturbances during a 1-month time period. The PSQI was translated into Chinese by Liu et al. (81). The instrument contains 19 items assessing seven components: subjective sleep quality, sleep latency, sleep duration, habitual sleep efficiency, sleep disturbances, use of sleep medication, and daytime dysfunction. The seven component scores are summed to produce a global PSQI score, where the total score ranges from 0 to 21 points (0 to 3 points for each component), with higher scores indicating worse sleep quality. The cut-off value for sleep disturbance is 7 (81). The Cronbach’s alpha of the PSQI was 0.71 in the present study.

#### International Positive and Negative Affect Scale Short-Form (I-PANAS-SF)

Positive affect and negative affect were assessed using the International Positive and Negative Affect Scale Short-Form (I-PANAS-SF) (82). The I-PANAS-SF comprises 10 items originating from the Positive and Negative Affect Schedule (PANAS) (61), and was translated into Chinese by Huang, Yang and Ji (83). NA and PA have five items respectively answered on a 5-point scale from 1 (“*not at all”*) to 5 (“*extremely”*). A higher NA total score indicated more negative affect or the extent to which the individual feels aversive mood states and general distress, whereas a higher PA total score indicates more positive affect or the extent to which the individual feels enthusiastic, active, and alert. The Cronbach’s alpha of the I-PANAS-SF was 0.72, for PA was 0.71, and for NA was 0.81 in the present study.

#### Chinese Trait-State Fear of Missing Out Scale (T-SFoMOSC)

The 12-item Trait-State Fear of Missing Out Scale (T-SFoMOS) was developed by Wegmann et al. (4) and assesses fear of missing out across two domains (i.e., trait-FoMO and state-FoMO). The first five items reflect trait-FoMO, whereas the remaining seven items are state-FoMO. Each item is rated on a 5-point Likert scale from 1 (“*totally disagree”*) to 5 (“*totally agree”*). Higher total scores represent a higher level of FoMO. The Cronbach’s alpha value of the trait-FoMO and state-FoMO were 0.82 and 0.81, respectively. In the present study, using the T-SFoMOSC, the Cronbach’s alpha for the total scale was 0.82, 0.78 for the trait-FoMO, and 0.81 for state-FoMO.

#### Mobile Phone Addiction Index (MPAI)

Smartphone addiction was assessed using the Mobile Phone Addiction Index (MPAI) (84), which was translated into Chinese (85) and is used widely in Chinese contexts (86, 87). The MPAI contains 17 items assessing four domains: inability to control craving, feeling anxious and lost, withdrawal/escape, and productivity loss. Each item is responded to from 1 (“*not at all”*) to 5 (“*always”*). Final scores were summed and higher total scores reflect higher levels of smartphone addiction. The Cronbach’s alpha of the MPAI was 0.86 in the present study.

### Procedure

The survey was conducted in three universities [Gannan Medical University (542 participants), Jiangxi University of Science and Technology (418 participants), and Jinzhou Medical University (298 participants)] in the two provinces of China from October 2019 to November 2019. Participants received a detailed explanation of the study’ purpose and completed the survey, and participants received course credits in mental health education classes. Four self-report scales including the PQSI, the I-PANAS-SF, the T-SFoMOS, and the MPAI were completed in approximately 10 min.

### Statistical Analysis

SPSS 20 was used for the present study analysis. The skewness and kurtosis levels were examined for data distribution (with a skewness cut-off of 2.0 and kurtosis cut-off of 7.0) (88). Descriptive analysis of the sample’ characteristics was conducted by means, standard deviations, and frequency analysis. A chi-square test was utilized for analyzing the gender difference in sleep disturbance. Pearson correlation tests were applied among all variables. The series of multiple mediation models of positive/negative affect on sleep quality *via* trait-FoMO/state-FoMO and smartphone addiction were tested using Model 6 of Hayes’s PROCESS tool (89).

### Ethics

The study was approved by the Ethics Committee of Gannan Medical University, and was carried out in accordance with the requisite ethical standards (e.g., the Helsinki declaration). Written informed consent was obtained from all participants.

## Results

### Descriptive Statistics and Correlation Analyses

Descriptive statistics are displayed in **Table 1**. Data were regarded as normal distribution due to the maximum values of skewness (< 2) and kurtosis (< 7). PA was significantly positively associated with state-FoMO (*r* = 0.09, *p* <.01), but significantly negatively associated with smartphone addiction (*r* = -0.06, *p* <.05) and poor sleep quality (*r* = -0.09, *p* <.01). PA was not significantly associated with the total scores of FoMO (*r* = 0.05, *p* = 0.110) and trait-FoMO (*r* = -0.02, *p* = 0.509). NA was significantly positively associated with trait-FoMO (*r* = 0.26, *p* <.01), state-FoMO (*r* = 0.16, *p* <.01), smartphone addiction (*r* = 0.31, *p* <.01), and poor sleep quality (*r* = 0.42, *p* <.01). Trait-FoMO was significantly positively associated with smartphone addiction (*r* = 0.33, *p* <.01) and poor sleep quality (*r* = 0.23, *p* <.01). State-FoMO was significantly positively associated with smartphone addiction (*r* = 0.42, *p* <.01) and poor sleep quality (*r* = 0.19, *p* <.01). Smartphone addiction was also significantly positively associated with poor sleep quality (*r* = 0.32, *p* <.01).

**Table 1:** Descriptive statistics and correlation analysis of the study variables.

		*M*	*SD*	Skewness	Kurtosis	1	2	3	4	5	6	7
1.	Sleep quality	5.26	2.29	0.279	-0.051	1.00						
2.	Positive affect	14.12	3.13	-0.206	0.504	-0.09**	1.00					
3.	Negative affect	10.69	3.37	0.389	-0.176	0.42**	0.07*	1.00				
4.	FoMO	29.07	6.95	-0.160	0.151	0.25**	0.05	0.24**	1.00			
5.	Trait-FoMO	12.19	3.63	0.008	-0.170	0.23**	-0.02	0.26**	0.79**	1.00		
6	State-FoMO	16.88	4.63	0.121	-0.007	0.19**	0.09**	0.16**	0.88**	0.41**	1.00	
7	Smartphone addiction	42.09	10.24	0.052	-0.116	0.32**	-0.06*	0.31**	0.45**	0.33**	0.42**	1.00

∗∗p < .01, ∗p < .05.

### The Prevalence of Sleep Disturbance

Based on 7 as the threshold value for the PQSI (81), of the 1,164 participants, 978 (84.02%) reported normal sleep quality and 186 (15.98%) reported sleep disturbance. The numbers of males (18.38%, male 506) and females (14.13%, female 658) with sleep disturbance were both 93. There was no significant difference between gender (χ^2^ = 3.53, *p* = 0.06).

### Testing of the Mediation Effects From PA to Sleep Quality

The series multiple mediation effects of PA on sleep quality *via* trait-FoMO/state-FoMO and smartphone addiction were tested using Model 6 of Hayes’ PROCESS tool. Gender and age were regarded as control variables in the present study. As shown in **Table 2**, the total mediation effects of PA on sleep quality *via* trait-FoMO and smartphone addiction (*β* = -0.018, 95%CI: -0.043, 0.006) as well as state-FoMO and smartphone addiction (*β* = -0.009, 95%CI: -0.033, 0.014) were both non-significant, whereas the direct effects of PA on sleep quality (*β* = -0.062, 95%CI: -0.114, -0.010; *β* = -0.070, 95%CI: -0.122, -0.018) were both significant. In the serial mediation model of trait-FoMO and smartphone addiction, the relationship between PA and poor sleep quality was not mediated by trait-FoMO (*β* = -0.003, 95%CI: -0.012, 0.006) or smartphone addiction (*β* = -0.014, 95%CI: -0.032, 0.003). However, in the serial mediation model of state-FoMO and smartphone addiction, the relationship between PA and poor sleep quality was mediated by smartphone addiction (*β* = -0.024, 95%CI: -0.042, -0.007), but not state-FoMO (*β* = 0.006, 95%CI: 0.000, 0.015).

**Table 2:** Total effect, direct effect, and mediation effects of PA/NA on sleep quality *via* trait-FoMO/state-FoMO and smartphone addiction.

Path (PA➔trait-FoMO)	B	SE	95%CI	Path (PA➔state-FoMO)	B	SE	95%CI
Total effect	-0.079	0.028	-0.134, -0.024	Total effect	-0.079	0.028	-0.134, -0.024
Direct effect	-0.062	0.026	-0.114, -0.010	Direct effect	-0.070	0.027	-0.122, -0.018
Total mediation effect	-0.018	0.012	**-0.043, 0.006**	Total mediation effect	-0.009	0.012	**-0.033, 0.014**
Ind1	-0.003	0.004	-0.012, 0.006	Ind1	0.006	0.004	0.000, 0.015
Ind2	-0.014	0.009	-0.032, 0.003	Ind2	-0.024	0.009	-0.042, -0.008
Ind3	-0.002	0.003	-0.007, 0.004	Ind3	0.008	0.004	0.000, 0.017
Path (NA➔trait-FoMO)	B	SE	95%CI	Path (NA➔state-FoMO)	B	SE	95%CI
Total effect	0.414	0.027	0.362, 0.466	Total effect	0.414	0.027	0.362, 0.466
Direct effect	0.336	0.028	0.282, 0.391	Direct effect	0.348	0.027	0.295, 0.402
Total mediation effect	0.078	0.012	**0.055, 0.103**	Total mediation effect	0.066	0.011	**0.046, 0.089**
Ind1	0.021	0.008	0.006, 0.037	Ind1	0.012	0.005	0.002, 0.024
Ind2	0.045	0.009	0.028, 0.063	Ind2	0.044	0.009	0.027, 0.062
Ind3	0.013	0.003	0.007, 0.019	Ind3	0.011	0.003	0.006, 0.017
C1	-0.024	0.013	-0.050, 0.002	C1	-0.032	0.012	-0.056, -0.009
C2	0.008	0.009	-0.009, 0.026	C2	0.001	0.006	-0.011, 0.014
C3	0.032	0.008	0.017, 0.049	C3	0.033	0.008	0.018, 0.049
Effect ratio (%)	19.1			Effect ratio (%)	16.2		

Confidence intervals for effects are bias corrected based on 5,000 bootstrap samples. a1. PA/NA to Trait-FoMO/State-FoMO, a2. PA/NA to smartphone addiction, b1. Trait-FoMO/State-FoMO to sleep quality, b2. Smartphone addiction to sleep quality, d1. Trait-FoMO/State-FoMO to smartphone addiction. Ind1 = PA/NA➔Trait-FoMO/State-FoMO➔Sleep quality, Ind2 = PA/NA➔Smartphone addiction➔Sleep quality, Ind3 = PA/NA➔Trait-FoMO/State-FoMO➔Smartphone addiction➔Sleep quality. CI = Ind1 minus Ind2, C2 = Ind1 minus Ind3, C3 = Ind2 minus Ind3. Effect ratio = Total mediation effect/Total effect. Bold values are highlighted to show where the 95% confidence interval (CI) contains zero, then the effect will not be significant at the 0.05 level..

### Testing of the Mediation Effects From NA to Sleep Quality via Trait-FoMO and Smartphone Addiction

**Figure 2:**
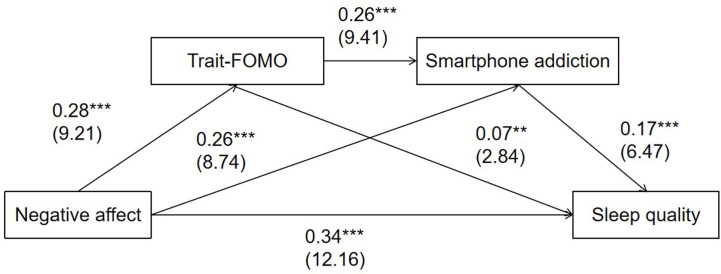
The serial multiple mediation model of fear of missing out and smartphone addiction on negative affect and sleep quality (n=1164). Coefficients are standardized and *t*-statistics are in parentheses. ***p < .001; **p < .01.

The serial multiple mediation effects of NA on sleep quality *via* trait-FoMO/state-FoMO and smartphone addiction were also examined and gender and age were also regarded as control variables. As **Figure 2** shows, NA was positively associated with trait-FoMO (*β* = 0.28, *t* = 9.21, *p* <.001) and smartphone addiction (*β* = 0.26, *t* = 8.74, *p* <.001). Trait-FoMO was positively associated smartphone addiction (*β* = 0.26, *t* = 9.41, *p* <.001) and poor sleep quality (*β* = 0.07, *t* = 2.84, *p* <.01). Smartphone addiction was positively associated with poor sleep quality (*β* = 0.17, *t* = 6.47, *p* <.001). In addition, the total indirect effect of NA and sleep quality *via* Trait-FoMO and smartphone addiction was significant (*β* = 0.078, 95%CI: 0.055, 0.103), as well as the direct effect (*β* = 0.336, 95%CI: 0.282, 0.391) and total effect (*β* = 0.414, 95%CI: 0.362, 0.466). Three indirect paths from NA to sleep quality *via* Trait-FoMO (*β* = 0.021, 95%CI: 0.006, 0.037), smartphone addiction (*β* = 0.045, 95%CI: 0.028, 0.063), and Trait-FoMO and smartphone addiction (*β* = 0.013, 95%CI: 0.007, 0.019), were significant respectively. Moreover, differences in the three paths were tested using Model 6 of the PROCESS tool. A significant difference (*β* = 0.032, 95%CI: 0.017, 0.049) between Path 2 (NA on sleep quality *via* smartphone addiction) and Path 3 (NA on sleep quality *via* trait-FoMO and smartphone addiction) was found (**Table 2**).

### Testing of the Mediation Effects From NA to Sleep Quality via State-FoMO and Smartphone Addiction

**Figure 3:**
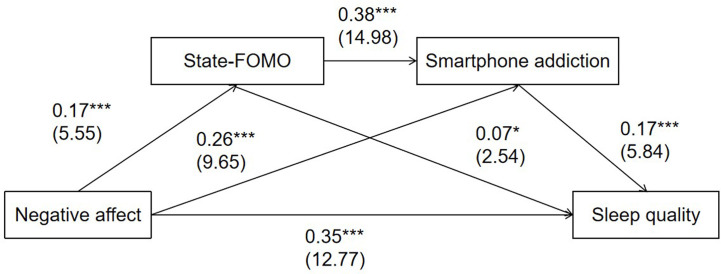
The serial multiple mediation model of fear of missing out and smartphone addiction on negative affect and sleep quality (n=1164). Coefficients are standardized and *t*-statistics are in parentheses. ***p < .001; *p < .05.

NA was positively associated with state-FoMO (*β* = 0.17, *t* = 5.55, *p* <.001) and smartphone addiction (*β* = 0.26, *t* = 9.65, *p* <.001). State-FoMO was positively associated with smartphone addiction (*β* = 0.38, *t* = 14.98, *p* <.001) and poor sleep quality (*β* = 0.07, *t* = 2.54, *p* <.05). Smartphone addiction was positively associated with poor sleep quality (*β* = 0.17, *t* = 5.84, *p* <.001) (**Figure 3**). In addition, the total indirect effect from NA to sleep quality *via* state-FoMO and smartphone addiction was significant (*β* = 0.066, 95%CI: 0.046, 0.089), as well as the direct effect (*β* = 0.348, 95%CI: 0.295, 0.402) and total effect (*β* = 0.414, 95%CI: 0.362, 0.466). Three indirect paths from NA to sleep quality *via* state-FoMO (*β* = 0.012, 95%CI: 0.002, 0.024), smartphone addiction (*β* = 0.044, 95%CI: 0.027, 0.062), and state-FoMO and smartphone addiction (*β* = 0.011, 95%CI: 0.006, 0.017) were all significant. Moreover, differences in the three paths were tested using Model 6 of the PROCESS tool. Significant differences between Path 1 (NA on sleep quality *via* state-FoMO) and Path 2 (NA on sleep quality *via* smartphone addiction) (*β* = -0.032, 95%CI: -0.056, -0.009), as well as Path 2 (NA on sleep quality *via* smartphone addiction) and Path 3 (NA on sleep quality *via* state-FoMO and smartphone addiction) (*β* = 0.033, 95%CI: 0.018, 0.049) were found. The proportion of total effect from NA to sleep quality *via* trait-FoMO and smartphone addiction was 19.1% and *via* state-FoMO and smartphone addiction was 16.2%.

## Discussion

The present study reported the prevalence of sleep disturbance and the mediation effects of positive and negative affect on sleep quality *via* trait-FoMO/state-FoMO and smartphone addiction among Chinese university students. The prevalence of sleep disturbance was 15.98%, which finding is similar to previous findings among Chinese samples (13.93%–15.8%) (29, 69, 70).

NA was positively associated with state-FoMO and trait-FoMO, whereas there was a positive correlation between PA and state-FoMO [e.g., “When I have a good time it is important for me to share the details online (e.g. updating status)”]. This may be because individuals with high state-FoMO like to share their enjoyment, interests, achievements with others anywhere at any time. As Przybylski et al. noted, individuals who experience high FoMO have mixed feelings [i.e., positive affect (excitement and joy) and negative affect (fear and anxiety)] when they use social media (1).

The correlation coefficient between FoMO and smartphone addiction (*r* = 0.42) was similar to study samples from other countries (ranging from 0.40 to 0.60) (11, 18, 90–92). This indicates that the impact of FoMO and excessive smartphone use are arguably ubiquitous in the global context (93–97).

NA was significantly positively associated with FoMO and smartphone addiction, which is consistent with other findings (18, 98). Elhai et al. reported that NA (i.e., depression and anxiety) may impact on smartphone addiction *via* FoMO (91), which was also consistent with the finding’s here. However, depression and interpersonal sensitivity on internet-communication disorder (ICD) *via* avoidance expectancies and state-FoMO have been found, but not for trait-FoMO (4). This perhaps indicates FoMO is a more complex construct. According to self-determination theory (SDT) (99), FoMO is considered as “a phenomenon of self-regulatory limbo arising from a situational or chronic deficit in psychological need satisfaction” (1). Consequently, FoMO was described as “a relatively stable individual characteristic, representing the general fear of a person of missing out on something” (i.e., trait-FoMO) (4). On the contrary, state-FoMO is considered important in the context of utilizing social media, where messages are updated and exchanged. State-FoMO even may increase general trait-FoMO (4). Based on the Brand et al.’s theory of specific internet addiction, a person’s core characteristics (e.g., depression and social anxiety) and personality (e.g., stress vulnerability, self-esteem, self-efficacy) predict specific cognitions, consequently causing different types of internet addiction (e.g., smartphone addiction and internet-communication addiction) (100, 101). In terms of interaction in the person-affect-cognition-execution (I-PACE) model (64), State-FoMO may represent a specific cognition, which mediates individual core characteristics (NA) and smartphone addiction, whereas trait-FoMO being a dispositional trait, may develop state-FoMO and specific internet cognition (4).

Results also showed that PA and NA both predicted sleep quality, and that NA had a more prominent influence than PA. These results suggest that regulation of NA for sleep disturbance therapy may be more successful than improvement of PA (73). Therefore, treatments targeting a reduction of NA may decrease insomnia symptoms and improve sleep quality (77). The mediation effects of NA on sleep quality *via* trait-FoMO/state-FoMO and smartphone addiction were statistically significant. However, the total indirect effects of PA on sleep quality *via* trait-FoMO/state-FoMO and smartphone addiction were not found. Moreover, smartphone addiction had higher mediation effect from NA to sleep quality. The relationship between NA, smartphone addiction, and sleep quality was also consistent with findings from a previous study (79).

Smartphone use may be pleasurable and exciting in the early stage when individuals communicate with others or engage in other activities (i.e., individuals experience positive affect). However, for a small minority of individuals, excessive smartphone use can trigger greater negative affect, such as irritable, anxiety, and depression, which may lead to smartphone addiction, and disturb individuals’ sleep quality. Some studies have reported that negative events impacting on sleep quality mainly included bad dormitory conditions (e.g., noisy roommates, snoring, gaming, lighting, mosquitos), interpersonal conflict, academic pressure, freshmen maladjustment, as well as romantic relationship problems) (102, 103). Due to specific characteristics of smartphones (e.g., the many different types of applications), smartphones can be considered as tools that provide self-comfort and satisfy basic need of communication with others in a timely way (i.e., individuals, especially females prefer texting, blogging and chatting *via* smartphone) (104–106). Consequently, all kinds of messages from Chinese SNSs such as *WeChat* and *QQ* (e.g., different groups comprising class, grade, students’ union, different courses, etc.) can seriously influence their academic performance and daily lives among Chinese university students. Therefore, one possible explanation is that NA (triggered by negative events and all kinds of stress) may increase the level of FoMO, and leads to students constantly check their smartphones (so as not to miss out on what they perceive to be important information) which among some individuals may give rise to smartphone addiction, and subsequently deteriorating sleep quality. The mediation effect of FoMO from NA to sleep quality appears to be weaker than smartphone addiction. These finding suggest that FoMO is closely associated with social media use, whereas smartphone addiction may be related to more specific activities (e.g., smartphone gaming addiction, smartphone shopping addiction, smartphone gambling addiction, etc.).

FoMO and smartphone addiction may mediate the relationship between negative affect and sleep quality. The pressures from school, college or university may prevent face-to-face social activity but being bombarded with excessive information *via* smartphone, which could lead to greater negative affect and affect both FoMO and smartphone addiction, subsequently leading students to have poorer sleep. However, several limitations are present in the present study. First, all data were self-report which may result in some biases (e.g., social desirability and memory recall). The cross-sectional study from three universities was non-representative, and is unable to explain any causal relationships between the variables tested. Future studies should be performed using more representative samples and longitudinal designs to determine if there are causal relationships between the variables FoMO, smartphone addiction, positive and negative affect, and sleep quality. Owing to gender and age as control variables in the present study, gender differences were not examined and there may be differences in relation to smartphone addiction as well as the relationship between affect and smartphone addiction. Demographic variables may potentially impact mediation effect (e.g., gender and age). Therefore such variables should be examined in future research. Second, further research is required to more deeply explore the characteristics of trait-FoMO and state-FoMO (e.g., studies evaluating the difference between trait-FoMO and state-FoMO as mediators). Third, further research is necessary to explain the specific positive affect and negative affect factors (e.g., alert, anxiety, fear) responsible for the relationships studied here.

Overall, the results demonstrate the mediation effects of trait-FoMO/state-FoMO and smartphone addiction between negative affect and sleep quality. More specifically, smartphone addiction had higher effect size than the role of FoMO. Based on the findings, negative affect on sleep quality may be mediated *via* smartphone addiction and FoMO among Chinese university students.

## Data Availability Statement

The raw data supporting the conclusions of this article will be made available by the authors, without undue reservation.

## Ethics Statement

The studies involving human participants were reviewed and approved by the Ethics Committee of Gannan Medical University. Written informed consent to participate in this study was provided by the participants’ legal guardian/next of kin.

## Author Contributions

Conceived and designed the experiments: LL and ZN. Performed the experiments: LL and ZN. Analyzed the data: LL and MG. Contributed reagents/materials/analysis tools: SM. Wrote the paper: LL and ZN. Edited and contributed to the revised paper: MG.

## Funding

The Science Education Program Project ”Thirteenth Five-Year Plan” of Jiangxi Province 2020GX184, Doctor start-up fund of Gannan Medical University QD201819, Key project of Gannan Medical University ZD201838, The International Innovation Team of Jilin University 2019GJTD06.

## Conflict of Interest

The authors declare that the research was conducted in the absence of any commercial or financial relationships that could be construed as a potential conflict of interest.
